# Intelligent Electrochemical Point-of-Care Test Method with Interface Control Based on DNA Pyramids: Aflatoxin B1 Detection in Food and the Environment

**DOI:** 10.3390/foods12244447

**Published:** 2023-12-12

**Authors:** Wenqin Wu, Yizhen Bai, Tiantian Zhao, Meijuan Liang, Xiaofeng Hu, Du Wang, Xiaoqian Tang, Li Yu, Qi Zhang, Peiwu Li, Zhaowei Zhang

**Affiliations:** 1 Oil Crops Research Institute of Chinese Academy of Agricultural Sciences, Key Laboratory of Biology and Genetic Improvement of Oil Crops, Key Laboratory of Detection for Mycotoxins, National Reference Laboratory for Agricultural Testing (Biotoxin), Hubei Hongshan Lab, Wuhan 430062, China; 2 School of Bioengineering and Health, State Key Laboratory of New Textile Materials and Advanced Processing Technologies, Wuhan Textile University, Wuhan 430200, China

**Keywords:** intelligent POCT, aflatoxin B1, DNA pyramid, screen-printed gold electrode, electrochemical biosensor

## Abstract

Sensitive, intelligent point-of-care test (iPOCT) methods for small molecules like aflatoxin B1 (AFB1) are urgently needed for food and the environment. The challenge remains of surface control in iPOCT. Herein, we developed an electrochemical sensor based on the DNA pyramid (DNP), combining a smartphone, app, and mobile electrochemical workstations to detect AFB1. The DNP’s structure can reduce local overcrowding and entanglement between neighboring probes, control the density and orientation of recognition probes (antibodies), produce uniform and orientational surface assemblies, and improve antigen–antibody-specific recognition and binding efficiency. Simultaneously, the hollow structure of the DNP enhances the electron transfer capacity and increases the sensitivity of electrochemical detection. In this work, the biosensor based on DNP was first combined with electrochemical (Ec) iPOCT to simultaneously achieve ordered interface modulation of recognition probes and intelligent detection of AFB1. Under optimal conditions, we found a detection limit of 3 pg/mL and a linear range of 0.006–30 ng/mL (R^2^ = 0.995). Further, using peanut, soybean, corn, and lake water as complex matrices, it recorded recoveries of 82.15–100.53%, excellent selectivity, acceptable stability, and good reproducibility. Finally, this Ec iPOCT provides consistent results compared to the high-performance liquid chromatography-tandem mass spectrometry method.

## 1. Introduction

Mycotoxins are common and serious co-contaminants in food and the environment. *Aspergillus flavus* exists throughout the food production cycle, from the soil, seeds, germination, growth, harvesting, transportation, and processing to the food on the table; it also lives in the entire food chain from the environment to food. Aflatoxin B1 (AFB1) is a toxic metabolite secreted by Aspergillus that has been classified by The International Agency for Research on Cancer classified as Group Ⅰ and is one of the most potent hepatotoxins, carcinogens, teratogens, and mutagens [1,2]. AFB1 harms human health through the environment–plant–food–human cycle [3] via direct and indirect ingestion. Many countries have established relevant laws to regulate the content of AFB1 in food. For example, permissible levels set by China are 20 µg/kg (corn, peanuts), 10 µg/kg (rice), and 5 µg/kg (soybeans) [4]. In the United States, the Food and Drug Administration set a maximum limit of 20 μg/kg for aflatoxin in peanuts and their products, nuts, and dried fruits [4]. The maximum allowed level in the European Union is 5 µg/kg for rice and 2 µg/kg for peanuts [5]. In Korea and Japan, the maximum allowed level is 1 µg/kg [6]. Therefore, it is urgent to establish an accurate and sensitive point-of-care testing method to detect AFB1 to achieve the purpose of supervision.

Conventional AFB1 determination methods include thin-layer chromatography, chromatography–mass spectrometry [7,8], and enzyme-linked immunosorbent assay [9]. Despite their accuracy and precision, they require highly skilled operators, extensive equipment, and lengthy analysis time. Therefore, a rapid analytical method is essential for the detection of AFB1. Electrochemical biosensors have attracted great interest due to their simplicity, low cost, easy operation, fast response, high sensitivity, real-time on-site monitoring, ease of online access, and automation. However, controlling the density and orientation of the electrode’s recognition probes (antibodies or aptamers) is challenging in electrochemical immunoassay methods. Aptamers or antibodies are prone to surface crowding effects, non-specific adsorption, and intertwining of probes [10], which can seriously affect the specific recognition and binding efficiency of AFB1 and ultimately affect the detection performance and accuracy. To address this issue, bridge antibodies have been introduced to the assembly process [11], contributing to an overall change in antibody orientation. Besides, although nanostructured surfaces were designed to overcome the effects of the probe density and enhance sensitivity, they involved multiple steps and complex fabrication [12,13]. DNA pyramids (DNPs) have excellent structural stability, mechanical rigidity, specific orientation, and accurately controlled recognition units [14,15,16]. Previous studies have demonstrated that a DNP (edge length of 17 bp) can be synthesized from four 55 nt DNA strands in a single step. Each strand of the DNP includes three blocks that can hybridize with three other strands, forming rigid triangles of DNA helices as one face of the tetrahedron with two oligonucleotide terminals merging at a vertex. Each side of the DNP is separated by several unhybridized nucleotides to provide sufficient flexibility for bending [15]. DNPs can precisely control the size and vertex position and introduce functionalized sequences. Additionally, DNPs efficiently maintain the distance between probes, avoiding probe entanglement and improving the binding efficiency of targets [17]. Simultaneously, the DNP nanostructure can be covalently bonded on the surface of a gold electrode via the interaction between thiol groups on DNA strands and gold. This enables the formation of a self-assembled DNA nanostructured monolayer on the surface of gold electrodes, projecting the antibodies in an upright orientation. Moreover, the structure can enhance the overall packing of tetrahedrons by maintaining a theoretical spatial separation distance of at least 4 nm, reducing the entanglement between neighboring probes and the local overcrowding effect [17,18]. The hollow structure of the tetrahedron can increase the sensitivity of electrochemical detection by enhancing the electron transfer capacity and can be synthesized rapidly and cost-effectively. The feasibility of constructing high-performance sensors has been evidenced using DNPs as binding sites [18]. By designing DNPs with varied sizes for DNA anchoring to precisely regulate the density, inter-probe entanglement was decreased, resulting in improved sensor sensitivity [19]. However, while DNPs are widely used in medical fields (antitumor and anti-inflammatory treatment, etc.) [20], they are rarely used in detecting common pollutants in food and the environment.

Nowadays, smartphones are available to almost everyone and play an essential role in the POCT field due to their ubiquity, portability, and versatility [21,22,23]. Smartphones are widely used in POCT to detect target analytes (pesticides, heavy metal ions, antibiotics, pathogens, bacteria, and biomarkers) in food safety, environmental issues, clinical testing, etc. However, challenges in POCT remain: (1) matching between smartphones, apps, and devices; (2) device configuration portability; (3) sensitivity and linearity in complex matrices; and (4) intelligence in real applications. An electrochemical intelligent point of care testing (Ec iPOCT) provides advantages due to its low cost, compact size, and portable device [24]. The smartphone can be successfully developed into a detection system with 3D printing technology or other readers through the built-in self-programming detection app. Smartphone-based Ec iPOCT is essential for the on-site detection of analytes.

Herein, we designed a DNP as an interfacial regulatory substrate to overcome the intertwining of probes and the disadvantages in Ec iPOCT. The DNP was covalently bonded to the surface of the screen-printed gold electrode (SPGE) through the interaction between gold and the thiol groups on DNA strands to form Au–S bonds by three thiol group-modified vertices. This significantly improves the stability of the sensing probes, precisely controls the surface density of the biosensing interface, and noticeably increases the target accessibility. Combining the biosensor modified by DNP, smartphone, app, and portable electrochemical workstation, an Ec iPOCT platform was constructed to detect mycotoxin in a timely manner. The biosensor was characterized by differential pulse voltammetry (DPV) and electrochemical impedance spectroscopy (EIS), and the density of DNP on the SPGE surface was calculated. Then, we chose AFB1 as an example in complex food samples (peanut, soybean, and corn) and the environment (lake water). After optimization, the Ec iPOCT was extensively evaluated for its selectivity, repeatability, and reproducibility. The Ec iPOCT was validated by comparing results with those obtained via HPLC-MS/MS using peanut, soybean, corn, and lake water samples. This Ec iPOCT can be widely employed in food safety and environmental monitoring.

## 2. Materials and Methods

### 2.1. Materials and Reagents

N-hydroxy succinimide (NHS), 1-ethyl-3-(3-dimethyl aminopropyl), carbodiimide hydrochloride (EDC), bovine serum albumin (BSA), Tris base, tris(2-carboxyethyl) phosphine hydrochloride (TCEP), sodium hydrogen phosphate (Na_2_HPO_4_), potassium dihydrogen phosphate (KH_2_PO_4_), magnesium chloride hexahydrate (MgCl_2_·6H_2_O), potassium chloride (KCl), hydrochloric acid (HCl), Tween 20, Tris-(hydroxymethyl)aminomethane, ethylenediaminetetraacetic acid (EDTA), AFB1, aflatoxin B_2_ (AFB2), aflatoxin G1 (AFG1), aflatoxin G_2_ (AFG2), deoxynivalenol (DON), zearalenone (ZEN), ochratoxin A (OTA), and fumonisin B1 (FB1) were purchased from Sigma-Aldrich Trading Co., Ltd. (Shanghai, China). All purified and modified oligonucleotides were synthesized by Sangon Biotech Co., Ltd. (Shanghai, China); the sequence’s detailed base sequence is shown in Table S1. 2-(N-morpholino)ethanesulfonic acid (MES) buffer (pH 6.0; 0.1 mol/L) was purchased from Fuzhou Phygene Biotechnology Co., Ltd. (Fuzhou, Fujian, China).

Ultrapure water (18 MΩ, 25 °C) was obtained from a Milli-Q water system (Milli-Q, 18 MΩ·cm resistivity at 25 °C, Merck Millipore, Burlington, MA, USA). All solutions were prepared with ultrapure water.

### 2.2. Preparation and Characterization of DNP

The DNP structure was self-assembled using the methods described in previous reports [10,11]. The identical color sequences in four DNA strands (O1-SH, O2-SH, O3-SH, O4-COOH) were hybridized and formed the pyramid structure via C-G and A-T base complementarity (Table S1, Figure S1).

Two microliters of each of the four DNA strands, 6 μL of the TCEP buffer (30 mM, preparation process was provided in Contents S1), and 36 μL of TM buffer (20 mM Tris, 50 mM MgCl_2_, pH 8.0, preparation process is provided in Contents S1) were mixed. TCEP buffer breaks the thiol groups’ disulfide bridges and facilitates hybridization. The DNP structure was synthesized by heating the mixture to 95 °C for 2 min and then cooling it to 0 °C for 30 s. The DNP structure was characterized by 2% agarose gum electrophoresis and compared with the hybrid products obtained by different kinds of single-stranded DNA (simultaneous existence of one, two, or three DNA strands).

### 2.3. Construction and Characterization of Ec iPOCT

First, the biosensor was developed based on a modified screen-printed gold electrode (SPGE). Three microliters of the prepared DNP were modified on the freshly cleaned SPGE surface and incubated overnight at 25 °C. The DNP became anchored on the SPGE surface via strong thiol–Au bonds to form an Au–S bond by three thiol group modified vertices to significantly improve the stability of the sensing probes, precisely control the surface density of biosensing interface, and noticeably increase the target accessibility (Figure S1). Finally, a specific orientation pyramid self-assembled layer on the surface of the SPGE was obtained. The remaining carboxyl group on the vertex was bound to the AFB1 antibody. AFB1-BSA and AFB1 monoclonal antibodies (mAbs) were prepared in our lab [25]. Next, 3 μL of freshly prepared solution of 0.4 M EDC and 0.1 M NHS in 0.1 M MES buffer (pH 6.0) were pipetted onto the gold surface and left to react for 0.5 h at 37 °C. In this way, the carboxylic group on the top of the DNP was activated. After washing with 0.01 M PBS (pH 7.4), 3 μL mAb (10 µg/mL) were added to the SPGE at 37 °C for 2 h. Finally, the 0.1 *w*/*v*% BSA solution was added to block the non-specific binding sites. EIS measurements characterized the constructed biosensor. The density (*θ*) of DNP on the SPGE surface was calculated by Formula (1).

(1) $$\theta =1-\frac{Rct(a)}{Rct(b)}\times 100\%$$

where *Rct*(*a*) and *Rct*(*b*) represent the *Rct* values of the bare SPGE and the DNP/SPGE, respectively.

Second, the intelligent detection platform was constructed by connecting the biosensor, smartphone, app, and portable electrochemical workstation (Figure S2). The biosensor was the three-electrode system SPGE (DS 250AT), containing the working gold electrode (4 mm diameter) modified by the signal probe (Ab/DNP), auxiliary electrode (platinum), and reference electrode (silver). The smartphone was a Nova 5i Pro (Huawei, Shenzhen, China). The portable electrochemical workstation and app were the mobile Smart PalmSens 4 (The Netherlands) and PStrace 5.7 (Palmsens, The Netherlands) app, respectively. After the biosensor was connected to the portable Smart PalmSens 4, and the app had connected to the instrument through Bluetooth, the test was started, and the detection results of the biosensor could be obtained through the mobile phone app (PStrace 5.7).

### 2.4. Determination of AFB1

Three microliters of the AFB1 matrix solution (containing various concentrations) was added dropwise to the sensor based on mAb/DNP/SPGE and incubated at 37 °C for 1 h to ensure a competent immune response. Finally, DPV measurements of the SPGE biosensor were carried out in the 0.01 M PBS solution containing 0.1 M KCl and 5 mM [Fe(CN)_6_]^3−/4−^, which served as a redox probe. The corresponding DPV measurements were operated in the potential range from −0.2 V to 0.6 V with a scan rate of 0.1 V/s. The peak current change value (ΔI) of DPV was calculated via Equation (2):

ΔI = I_0_ − I_d_ (2)

where I_0_ and I_d_ are the peak current obtained in the blank solution and the different concentrations of AFB1, respectively.

### 2.5. Optimization of mAbs, pH, Incubation Temperature, and Time

To obtain high sensitivity for AFB1 detection, several analytical conditions were optimized, including the AFB1 antibody concentration, pH of the 0.01 M PBS electrolyte solution (5 mM [Fe(CN)_6_]^3−/4−^), incubation time, and incubation temperature.

The effect of AFB1 antibody concentrations between 2.5 and 20.0 µg/mL (2.5, 5.0, 7.5, 10.0, 12.5, 15.0, 17.5, 20.0 µg/mL) on the ΔI value for the detection of 50 ng/mL AFB1 was evaluated. The influence of pH (6.4, 6.8, 7.0, 7.4, 7.8, 8.0) on the electrochemical response was investigated. The incubation time (10, 20, 30, 40, 50, 60, 70, 80, 90 min) and incubation temperature (20, 25, 30, 35, 37, 40, 45, 50 °C) were optimized by the checkerboard method by comparing the ΔI value of the SPGE biosensor for 10 ng/mL AFB1.

### 2.6. Evaluation of the Ec iPOCT

The linear ranges and LODs were calculated via the calibration curve. The AFB1 calibration curve was established based on the DPV measurement for different concentrations of AFB1 (0 pg/mL, 0.04 pg/mL, 0.2 pg/mL, 0.6 pg/mL, 2 pg/mL, 6 pg/mL, 10 pg/mL, 0.1 ng/mL, 1 ng/mL, 5 ng/mL, 10 ng/mL, 20 ng/mL, 30 ng/mL, 40 ng/mL). Each data point in the standard curve was repeated three times. LODs were calculated by LOD = X + 3SD, in which SD and X were the standard deviation values and the average concentration of 21 repeated experiments in a blank solution, respectively [26,27].

Reproducibility, recovery, and repeatability were used to evaluate the performances of the SPGE biosensor. The negative peanut and soybean samples were spiked to 0, 2, 20 ng/mL, the negative lake water samples were spiked to 0, 2, 15 ng/mL, and the positive corn sample was spiked to 0, 2 ng/mL. The spiked experiments calculated recoveries.

To evaluate the selectivity of the Ec iPOCT biosensor for AFB1, other interferences, such as 10 ng/mL AFB2, AFG1, AFG2, DON, ZEN, OTA, FB1, and their mixture (10 ng/mL of AFB1 containing 10 ng/mL of AFB2, AFG1, AFG2, DON, ZEN, OTA, FB1) were tested under identical conditions. The DPV ΔI value was recorded and compared in the presence of these interferences.

The stability of one biosensor was carried out by comparing the current responses of the SPGE biosensor for 10 ng/mL AFB1, corresponding to 11 repeated tests. The reproducibility between different electrodes was also compared by performing DPV measurements of 10 ng/mL AFB1 with six electrodes.

### 2.7. Validation of Ec iPOCT Results by Comparing with HPLC-MS/MS Using Real Samples

The detection results were compared between the Ec iPOCT biosensor and HPLC-MS/MS using spiked real samples to verify the accuracy of the developed Ec iPOCT method. The samples and concentrations were the same as in the recovery experiment.

### 2.8. Sample Preparation

Samples (peanut, soybean, corn) were purchased from a local market in Wuhan, and the lake water was collected from Shahu in Wuhan, China. The homogenized samples (peanut, soybean, and corn) were spiked with AFB1 standard at different concentrations. Next, 10 mL of acetonitrile solution (*v*/*v*, 70%) was added to 2 g of spiked sample and extracted for 3 min by vortexing. After centrifugation (4000× *g*, 2 min), the supernatant was filtered (0.22 μm filter membrane) [28]. Then, 1.0 mL of supernatant was added to 3.0 mL of PBS, vortex mixed, and stored at 4 °C as the test solution. After filtering through a water filter membrane, the lake water extraction method above was used for pre-treatment extraction.

## 3. Results and Discussion

### 3.1. Preparation and Characterization of DTNs

**Figure 1:**
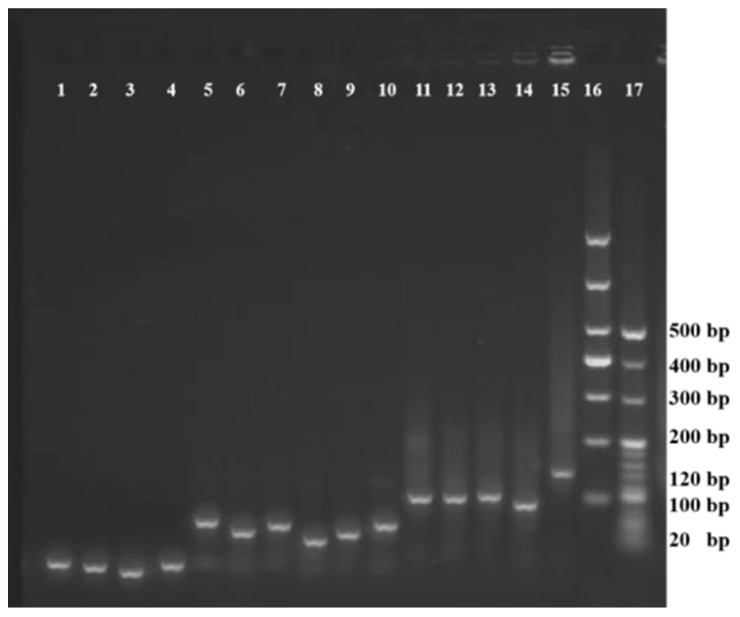
The 2% agarose gum electrophoresis characterization of the DNA sequences involved in TCEP synthesis contain single-stranded DNA (1, 2, 3, 4), two-strand (5, 6, 7, 8, 9, 10) or three-strand (11, 12, 13, 14) fragments, and DNP (15). DNA sequences involved in synthesis, 1: O1-SH, 2: O2-SH, 3: O3-SH, 4: O4-COOH, 5: O1-SH and O2-SH, 6: O1-SH and O3-SH, 7: O1-SH and O4-COOH, 8: O2-SH and O3-SH, 9: O2-SH and O4-COOH, 10: O3-SH and O4-COOH, 11: O1-SH, O2-SH, and O3-SH, 12: O1-SH, O2-SH, and O4-COOH, 13: O1-SH, O3-SH, and O4-COOH, 14: O2-SH, O3-SH, and O4-COOH, 15: O1-SH, O2-SH, O3-SH, and O4-COOH (DNP), 16: DL1000 NDA marker, 17: 20 bp DNA ladder.

The 2% agarose gum electrophoresis characterization of DNP and the various strand combination groups (simultaneous existence of one, two, or three DNA strands) are shown in Figure 1. The DNP (column 15) shifted slower than the combinations of three strands (columns 11, 12, 13, 14), two strands (columns 5, 6, 7, 8, 9, 10), and the single-stranded DNA (column 1, 2, 3, 4); the DNP migrated more slowly than the other combinations of two or three fragments. The relative size of DNP was 120 bp compared with the DL1000 NDA marker (column 16) and the 20 bp DNA ladder (column 17). This result agrees with previous reports [14,15]. The electrophoresis results indicated that the DNP was successfully synthesized.

### 3.2. Construction of the Pyramid DNA Nanostructure

Before fabricating the biosensor, we deactivated and cleaned the electrodes. Electrochemical measurement was carried out in 0.5 M H_2_SO_4_ to remove impurities from the surface of the SPGE. First, we sequentially applied 2 V potential for 5 s and −0.35 V for 10 s to the SPGE. The CV measurement was then performed via 5–10 cycles in 0.5 M H_2_SO_4_ solution (dropped on the electrode surface) under a scan rate of 0.1 V/s, with a potential range of −0.3 to 1.5 V.

**Figure 2:**
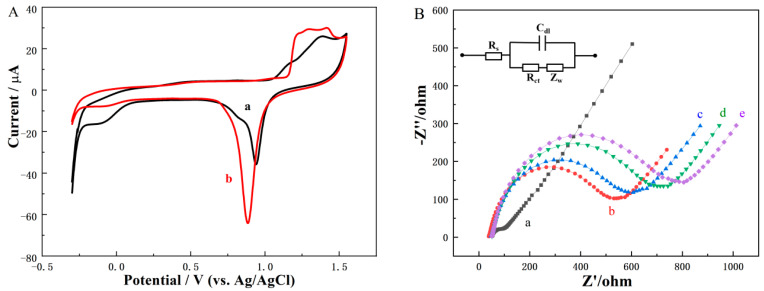
(**A**) CV curves of the bare SPGE before (a) and after (b) activation by electrochemistry in 0.5 M H_2_SO_4_. (**B**) EIS curves at different modification stages: (a) bare SPGE, (b) DNP/SPGE, (c) mAbs/DNP/SPGE, (d) mAbs/BSA/DNP/SPGE, (e) mAbs/BSA/DNP/SPGE reacted with 1 ng/mL AFB1; the working solution was 5 mM [Fe(CN)_6_]^3−/4−^ with 0.1 M KCl.

We then compared the CV curves of the bare SPGE before/after activation using electrochemistry in 0.5 M H_2_SO_4_ (Figure 2A). Curve b shows a typical cyclic voltammogram of pure gold [29]. Three continuous small oxidation peaks of gold are visible at 1.22, 1.29, and 1.42 V. A characteristic reduction peak of gold oxides is at 0.8 V, indicating the SPGE electrode was cleaned and activated and can be used after modification.

EIS was employed to characterize the assembling process by detecting the interface value of the electrochemical immunosensor during different modification steps. The EIS measurements were carried out in the frequency range of 0.1 Hz–100 kHz and a voltage amplitude of 5 mV at the formal potential of 0.21 V in 5 mM [Fe(CN)_6_]^3−/4−^ containing 0.1 M KCl. Figure 2B shows the EIS curves of other modified sensors to investigate the stepwise assembly process of the electrode: the bare SPGE (curve a), DNP/SPGE (curve b), BSA/DNP/SPGE (curve c), mAbs/BSA/DNP/SPGE (curve d), and mAbs/BSA/DNP/SPGE reacted with 1 ng/mL AFB1 (curve e).

In Figure 2B, the bare SPGE exhibits a small semicircle (curve a, Rct = 94.5 Ω), which indicates a good electron transfer on the plain SPGE surface. The semicircle diameter of the DNP/SPGE was significantly increased compared with that of SPGE (curve b, Rct = 530.8 Ω), indicating that the DNP was successfully modified to the SPGE surface. The density of DNP modified on the SPGE was calculated by Formula (1). The calculated θ value was 82.2%, exhibiting a relatively high coverage of DNP on the SPGE surface [30]. The immobilization of AFB1 mAbs onto the DNP/SPGE increases the impedance (curve c, Rct = 587.5 Ω), proving the antibody was successfully immobilized on SPGE. Subsequent blocking of other sites with BSA increased the impedance (curve d, Rct = 673.5 Ω). When the mAbs/BSA/DNP/SPGE reacted with 1 ng/mL AFB1, the Rct value increased to 744.5 Ω, indicating that the mAbs successfully captured the AFB1.

### 3.3. Optimization Ec iPOCT

**Figure 3:**
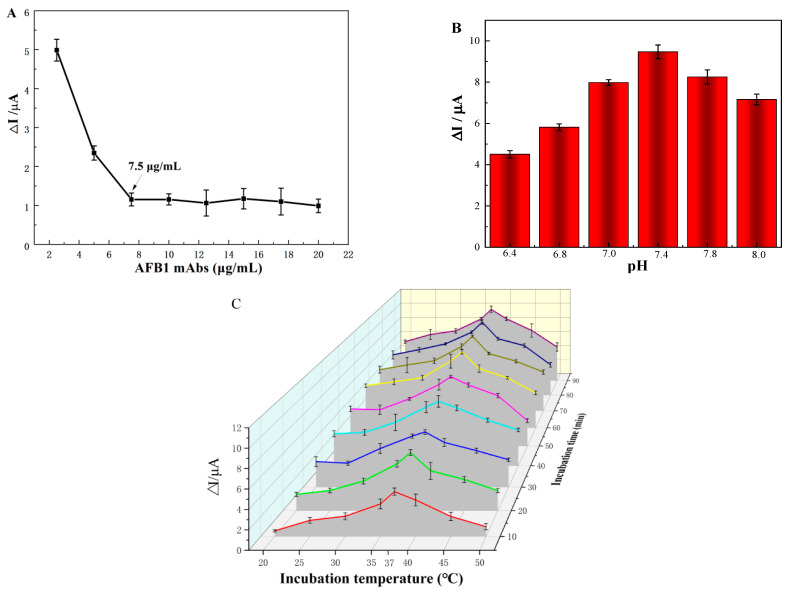
(**A**) optimization of the antibody concentration (n = 3), the ΔI value change trend of the biosensor reaction with 50 ng/mL AFB1; optimization of (**B**) the electrolyte solution pH (n = 3), (**C**) incubation temperature, and incubation time (n = 3).

The AFB1 antibodies immobilized on the modified SPGE electrode have an essential effect on the sensitivity of the immunosensor because they provide binding sites for AFB1. Figure 3A shows the effect of AFB1 antibody concentrations between 2.5 and 20 µg/mL on the ΔI for detecting 50 ng/mL AFB1. The ΔI increased sharply when the antibody concentration was below 7.5 µg/mL and plateaued after 7.5 µg/mL due to antibody saturation. Therefore, 7.5 µg/mL AFB1 antibodies was chosen as the optimum concentration.

The pH of the electrolyte solution was a crucial parameter that affected the sensitivity of the immunosensor. Electrolyte solutions with different pH values (6.4–8.0) were investigated. In Figure 3B, as the pH increased from 6.4 to 7.4, the ΔI current gradually rose, reaching a maximum value at pH 7.4, because this pH promoted the active conformation of the antibody to interact with targets, while higher or lower pH values affected the structure of the antibody and even the effective binding of AFB1 [31]. Thus, the electrolyte solution with pH 7.4 was used as the final electrolyte solution.

The checkerboard method was used to optimize the incubation temperature and time simultaneously. Figure 3C shows the relationship between DPV ΔI values with incubation time and temperature. The ΔI increases gradually until 60 min and then plateaus, indicating that the antibody and antigen have been fully coupled to reach saturation. The ΔI increased rapidly, peaking at 37 °C and then decreased steeply thereafter. This could be attributed to the progressively strengthened antibody activity as the temperature increased from room temperature to 37 °C. The excessive temperature could denature or devitalize the antibodies. Finally, 37 °C and 60 min were the optimal incubation temperature and time for the antigen–antibody interaction.

### 3.4. Evaluation of Ec iPOCT

**Figure 4:**
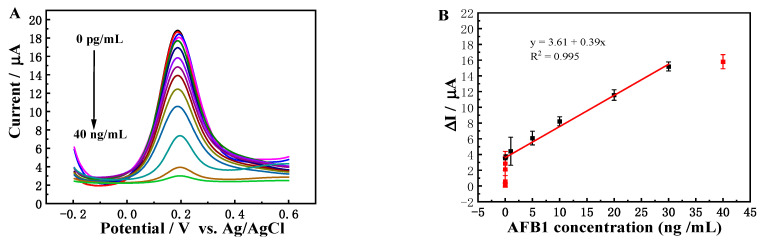
(**A**) DPV responses of the Ec iPOCT biosensor for different concentrations of AFB1 (0 pg/mL, 0.04 pg/mL, 0.2 pg/mL, 0.6 pg/mL, 2 pg/mL, 6 pg/mL, 10 pg/mL, 0.1 ng/mL, 1 ng/mL, 5 ng/mL, 10 ng/mL, 20 ng/mL, 30 ng/mL, 40 ng/mL); (**B**) corresponding calibration curve (n = 3).

Under the optimal conditions, the responses of the constructed mAbs/BSA/DNP/SPGE biosensor for AFB1 detection were recorded with DPV by varying the AFB1 concentration from 0 pg/mL to 40 ng/mL. The DPV ΔI value decreased with the increasing AFB1 concentration (Figure 4A) due to the formation of antigen–antibody immunocomplexes between AFB1 and antibodies impeding electron transport. Figure 4B presents the variation in DPV peak current change (ΔI = I_0_ − I_d_) and AFB1 concentration. The ΔI gradually increased with an increase in AFB1 concentration. The calibration plot displays an excellent linear relationship between ΔI and AFB1 concentration from 0.006 ng/mL to 30 ng/mL. The corresponding regression equation was ΔI = 3.61 + 0.39 C_AFB1_ (R^2^ = 0.995). The LOD was 0.003 ng/mL (S/N = 3).

Compared with previous reports (Table 1), the proposed Ec iPOCT biosensor has a desirable linear range and low LOD (10–67 times lower) [32,33]. The excellent properties of the DNP nanostructure endowed the SPGE surface with less overcrowding coverage and directly increased the analyte accessibility.

### 3.5. Selectivity, Stability, and Reproducibility of the Ec iPOCT Biosensor

**Figure 5:**
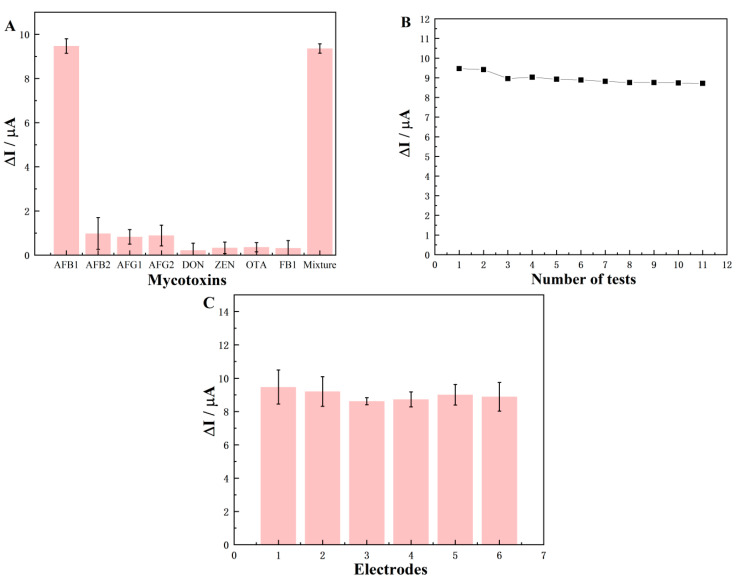
(**A**) electivity of the Ec iPOCT biosensor in the presence of different mycotoxins (10 ng/mL, n = 3). (**B**) Reproducibility of one biosensor; ΔI value of the Ec iPOCT biosensor reacted with 10 ng/mL AFB1, 11 repeated tests. (**C**) DPV peak current change of six biosensors for 10 ng/mL AFB1 (n = 3).

Figure 5A shows the selectivity of the proposed Ec iPOCT biosensor for detecting AFB1. Under the same condition, the ΔI value of the interferences (AFB2, AFG1, AFG2, OTA, DON, ZEN, FB1) was relatively negligible compared with that for AFB1, demonstrating the excellent selectivity of the Ec iPOCT biosensor. Figure 5B shows that the ΔI value of the Ec iPOCT biosensor retained 92.08% of its original value after 11 repeated tests, demonstrating the good stability of the proposed immunosensor (RSD = 2.93%, n = 11).

Figure 5C shows the DPV measurements for six of the same modified electrodes for 10 ng/mL AFB1 under the same conditions. The RSD was 7.87%, revealing excellent reproducibility.

### 3.6. Validation and Comparison with HPLC-MS/MS in Real Samples

The peanut, soybean, corn, and lake water samples were assessed to verify the practical application of the developed Ec iPOCT biosensor for real samples. The HPLC-MS/MS results revealed an AFB1 concentration of 15.43 ng/mL in the corn sample, while the peanut, soybean, and lake water samples were undetectable. Table 2 shows the spiked recovery test results, ranging from 82.15% to 100.53%, and the RSD, which varied from 1.15% to 5.42%. The AFB1 concentrations determined by the biosensor agreed well with the HPLC-MS/MS. These results illustrated that the developed immunosensor possesses excellent reliability and accuracy for detecting AFB1 in real samples.

## 4. Conclusions

In summary, this work constructed the Ec iPOCT platform, comprising the DNP biosensor, a smartphone, app, and portable electrochemical workstation that can be used for the mobile detection of AFB1. The Ec iPOCT biosensor exhibited a wide detection range (0.006–30 ng/mL, R^2^ = 0.995) and low LOD (3 pg/mL) in complex real samples. The biosensor successfully performed AFB1 detections in spiked peanut, soybean, corn, and lake water samples with recovery rates from 82.15% to 100.53%, and the RSD varied from 1.15% to 5.42%. In addition, the sensor has the advantages of excellent reproducibility (RSD was 7.87%, n = 6), selectivity, and good stability (RSD = 2.93%), retaining 92.08% of its original value after 11 repeated tests. These advantages stem from the surface control of the biosensor provided by the DNPs. The DNP structure can reduce local overcrowding and entanglement between neighboring probes, control the density and orientation of recognition probes (antibodies), produce uniform and orientational surface assemblies, and improve antigen–antibody-specific recognition and binding efficiency. Simultaneously, the hollow structure of the DNPs enhance the electron transfer capacity and increase the sensitivity of electrochemical detection. However, proper recognition probes, including antigens and antibodies of the target, must be developed and applied in the biosensor to achieve specific recognition and intelligent detection. This method provides technical support for AFB1 monitoring. The technique can be expanded to detect other common pollutants in food and the environment by altering the antigens and antibodies. This Ec iPOCT can be extensively employed in food and environmental safety. Other target analytes based on the biosensor will be prepared in the future.

## Figures and Tables

**Table 1:** Performance of the proposed Ec iPOCT biosensor compared with other amperometric sensors for detecting AFB1.

Immunoelectron	Sensitivity (μA/ng/mL)	Linear Range (ng/mL)	LOD (ng/mL)	Detection Method	Ref.
BSA/anti-AFB1/AuNPs/CHI/GCE	0.02	0.6–110	0.2	DPV	[32]
MWCNTs/RTILs/Ab/AFB1	/	0.1–10	0.03	EIS	[33]
BSA/anti-AFB1/CHI-AuNPs/Au microelectrode	1.20 0.03	0.1–1.0 1–30	0.06	DPV	[34]
CNTs/PDDA/Pd–Au	/	0.05–25	0.03	DPV	[35]
CS-AuNPs/gold microelectrode	0.72 0.06	0.2–2 2–30	0.12	CV	[36]
HRP/DTP/PANI/gold electrode	/	0.05–20	0.033	DPV	[37]
Ec iPOCT biosensor	/	0.006–30	0.003	DPV	This Work

**Table 2:** Recovery analysis of AFB1 in peanut, soybean, corn, and lake water samples by the proposed Ec iPOCT biosensor (n = 3).

Sample	Original (µg/kg)	Spiked (µg/kg)	Ec iPOCT Biosensor			HPLC-MS/MS ^b^ (µg/kg)
			Found ^b^ (µg/kg)	Recovery (%)	RSD (%)	
Peanut	ND ^a^	0	ND	/	2.09	ND
		2	1.81 ± 0.26	90.50	2.98	1.79 ± 0.73
		20	16.43 ± 0.89	82.15	5.12	17.20 ± 1.32
Soybean	ND	0	ND	/	2.06	ND
		2	1.73 ± 0.13	86.50	1.15	1.82 ± 0.04
		20	19.59 ± 1.26	97.95	1.81	19.42 ± 1.02
Corn	15.43 ± 0.23 ^c^	0	15.12 ± 0.86	/	3.47	15.43 ± 0.96
		2	17.24 ± 0.92	90.5	2.62	17.37 ± 1.24
Lake water	ND	0	ND	/	3.50	ND
		2	1.88 ± 0.06	94.00	4.33	1.86 ± 0.09
		15	15.08 ± 0.79	100.53	5.42	14.96 ± 0.68

^a^ ND: not detected, ^b^ Averages of three different samples, ^c^ Result via HPLC-MS/MS.

## Data Availability

Data is contained within the article.

## Supplementary Materials

The following supporting information can be downloaded at https://www.mdpi.com/article/10.3390/foods12244447/s1. Contents S1: Buffer solutions; Contents S2: Synthesis of DNA pyramid; Contents S3: Intelligent detection platform; Table S1: Oligonucleotide DNA sequences used to synthesize DNA pyramid (DNP); Figure S1: The synthesis steps of the DNP; Figure S2: The images of the portable electrochemical workstation, smartphone app, and portable integrated detection equipment.
