# Supplementary material for: Intelligent Electrochemical Point-of-Care Test Method with Interface Control Based on DNA Pyramids: Aflatoxin B1 Detection in Food and the Environment
Source: Foods. 2023 Dec 12;12(24):4447. doi: 10.3390/foods12244447 (PMC10743006; doi:10.3390/foods12244447)
Supplement: Supplementary file 1 [file foods-12-04447-s001.zip › foods-2762960-supplementary.pdf]

Supplementary Material

Intelligent Electrochemical Point-of-Care Test Method with Interface Control based on DNA Pyramids: Aflatoxin B1 Detection in Food and the Environment

Wenqin Wu [1], Yizhen Bai [1], Tiantian Zhao [1], Meijuan Liang [1], Xiaofeng Hu [1], Du Wang [1], Xiaoqian Tang [1], Li Yu [1], Qi Zhang [1], Peiwu Li [1], and Zhaowei Zhang [1,2,]*

1 Oil Crops Research Institute of Chinese Academy of Agricultural Sciences, Key Laboratory of Biology and Genetic Improvement of Oil Crops, Key Laboratory of Detection for Mycotoxins, National Reference Laboratory for Agricultural Testing (Biotoxin), Hubei Hongshan Lab, Wuhan, 430062, PR China

2 School of Bioengineering and Health, State Key Laboratory of New Textile Materials and Advanced Processing Technologies, Wuhan Textile University, Wuhan 430200, China

* Correspondence: zwzhang@whu.edu.cn; Tel.: +86-27-8671-1839

Contents

S1. Buffer solutions

S2. Synthesis of DNA pyramid

S3. Intelligent detection platform

S1. Buffer solutions

The buffer solutions employed in this study were as follows:

TCEP solution (30 mM): add 8.6 mg of TCEP to 1 mL of Milli-Q water. TCEP solution should be freshly prepared before use.

TM buffer (pH 8.0): mix 0.242 g of Tris base, 1.016 g of $MgCl_2 \cdot 6H_2O$ and 90 mL of Milli-Q water. Use HCl to adjust the solution to pH 8.0 and fill the solution to a final volume of 100 mL. Store the buffer at 4°C for at least 6 months.

PBS buffer (0.01 M): mix 8 g of NaCl, 0.2 g of KCl, 1.44g of Na_2HPO_4, 0.27 g of KH_2PO_4 and 800 mL of Milli-Q water. Use HCl to adjust the solution to pH 7.4 and fill the solution to a final volume of 1000 mL. Store the buffer at 4°C for at least 6 months.

PBST: add 5 mL of Tween 20 to 1 liter of 0.01 M PBS buffer. Store the buffer at 4°C for at least 1 month.

S2. Synthesis of DNA pyramid

Table S1. Oligonucleotide DNA sequences used to synthesize DNA pyramid (DNP)

**Name**	**Sequence**
O1-SH	5'-HS-C6-TTCAGACTTAGGAATGTGCTTCCCACGTAGTGTCGTTTGTATTGGACCCTCGCAT-3' (55 bases)
O2-SH	5'-HS-C6-TCAACTGCCTGGTGATAAAACGACACTACGTGGGAATCTACTATGGCGGCTCTTC-3' (55 bases)
O3-SH	5'-HS-C6-TATCACCAGGCAGTTGACAGTGTAGCAAGCTGTAATAGATGCGAGGGTCCAATAC-3' (55 bases)
O4-COOH	5'-COOH-TTTTTTTTTT-ACATTCCTAAGTCTGAAACATTACAGCTTGCTACACGAGAAGAGCCGCCATAGTA-3' (65 bases)

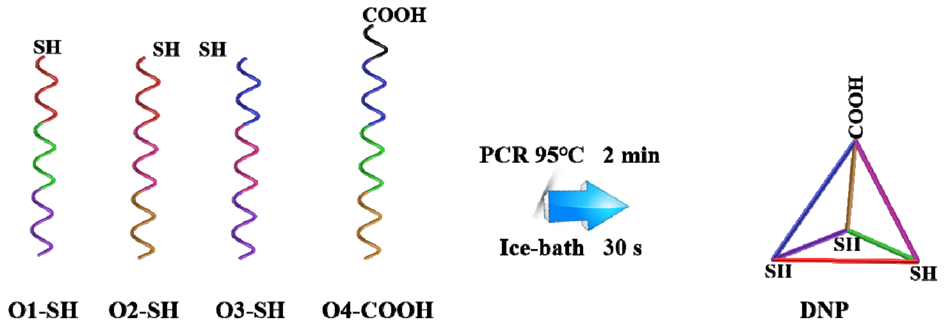

Figure S1. The synthesis steps of the DNP

S3. Intelligent detection platform

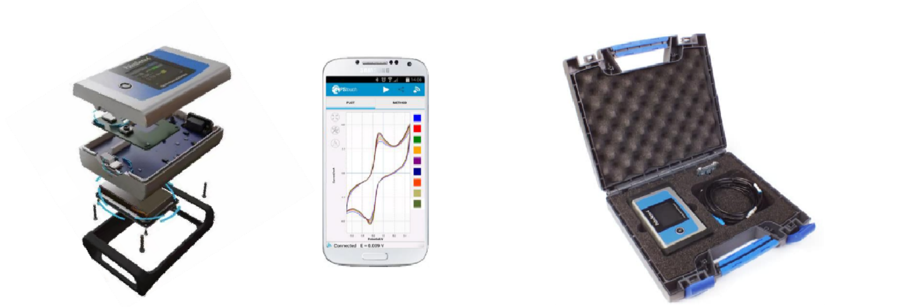

PalmSens 4 **Smartphone App** **Portable integrated testing equipment**

Figure S2. The images of the portable electrochemical workstation, smartphone app, and portable integrated detection equipment.
